# Integrating PPI datasets with the PPI data from biomedical literature for protein complex detection

**DOI:** 10.1186/1755-8794-7-S2-S3

**Published:** 2014-10-22

**Authors:** Zhi Hao Yang, Feng Ying Yu, Hong Fei Lin, Jian Wang

**Affiliations:** 1 College of Computer Science and Technology, Dalian University of Technology, Dalian, China

**Keywords:** Protein-protein interaction network, Protein complexes, Information extraction, Text mining

## Abstract

**Background:**

Protein complexes are important for understanding principles of cellular organization and function. High-throughput experimental techniques have produced a large amount of protein-protein interactions (PPIs), making it possible to predict protein complexes from protein-protein interaction networks. On the other hand, the rapidly growing biomedical literature provides a significantly large and readily available source of interaction data, which can be integrated into the protein network for better complex detection performance.

**Methods:**

We present an approach of integrating PPI datasets with the PPI data from biomedical literature for protein complex detection. The approach applies a sophisticated natural language processing system, PPIExtractor, to extract PPI data from biomedical literature. These data are then integrated into the PPI datasets for complex detection.

**Results:**

The experimental results of the state-of-the-art complex detection method, ClusterONE, on five yeast PPI datasets verify our method's effectiveness: compared with the original PPI datasets, the average improvements of 3.976 and 5.416 percentage units in the maximum matching ratio (MMR) are achieved on the new networks using the MIPS and SGD gold standards, respectively. In addition, our approach also proves to be effective for three other complex detection algorithms proposed in recent years, i.e. CMC, COACH and RRW.

**Conclusions:**

The rapidly growing biomedical literature provides a significantly large, readily available and relatively accurate source of interaction data, which can be integrated into the protein network for better protein complex detection performance.

## Background

Protein complexes are molecular aggregations of proteins assembled by multiple protein-protein interactions. Many proteins are functional only after they are assembled into a protein complex and interact with other proteins in this complex. These protein complexes can help us to understand the principles of cellular organization and function. High-throughput experimental techniques have produced a large amount of protein interactions, which makes it possible to uncover protein complexes from protein interaction networks. A protein interaction network can be modeled as an undirected graph, where vertices represent proteins and edges represent interactions between proteins. Protein complexes are groups of proteins that interact with one another, so they are usually dense sub-graphs in PPI networks. Various algorithms based on graph theory have been applied to identify protein complexes and functional modules from protein interaction networks, including CFinder [1], CMC [2], COACH [3], MCL [4], RRW [5] and ClusterONE [6].

At the same time, a number of databases, such as Gavin [7], Krogan [8], Collins [9], DIP [10], and BioGRID [11], have been created to store protein interaction information in structured and standard formats. These datasets were usually derived with different experimental techniques: the Collins, Krogan and Gavin datasets include the results of TAP tagging experiments only; the DIP dataset include the results of Y2H experiments; the BioGRID dataset contains a mixture of TAP tagging, Y2H and low-throughput experimental results. However, even for model species, only a fraction of true physical interactions are known [12,13] and experimental verification of all remaining potential interactions is unlikely in the near future [14]. On the other hand, the rapidly growing biomedical literature provides a significantly large and readily available supplemental source of PPI data for complex detection methods. What is more, since these data from biomedical literature are contributed by biologists and, therefore, relatively accurate, the integration of them into the existing PPI datasets can be hopeful for better complex detection performance.

Our work aims to quantifying the contribution of PPI data from biomedical literature as a supplement to the existing PPI datasets. In this paper, we present an approach of integrating PPI datasets with the PPI data from biomedical literature for protein complex detection. The approach applies a sophisticated natural language processing system, PPIExtractor [15], to extract new interactions from biomedical literature. These data are then integrated into the PPI datasets for protein complex detection. The experimental results on several PPI datasets show that in most cases the performances of some state-of-the-art protein complex detection methods are improved through the integration of protein-protein interactions and the PPI data extracted from literature.

## Methods

### Extracting PPIs with PPIExtractor

In this work, we apply the PPIExtractor system to extract PPI data from biomedical literature, which are then integrated into the protein network for protein complex detection.

Among the popular machine learning approaches to extracting PPIs from biomedical literature, kernel-based methods including tree kernels [16], shortest path kernels [17], and graph kernels [18] have been proposed for PPIs extraction. Kernel-based methods retain the original representation of objects and use the object in algorithms only via computing a kernel function between a pair of objects. However, each kernel utilizes a portion of the structures to calculate useful similarity. The kernel cannot retrieve the other important information that may be retrieved by other kernels.

In previous work, we presented PPIExtractor to automatically extract protein-protein interactions from biomedical literature. PPIExtractor is a multiple kernels learning based system which combines the feature-based, convolution tree and graph kernels to extract PPIs. The combined kernel can reduce the risk of missing important features, yielding new useful similarity measures. More specifically, the weighted linear combination of individual kernel used instead of assigning the same weight to each individual kernel is experimentally proven to contribute to the performance improvement. Experimental evaluations show that PPIExtractor can achieve state-of-the-art performance on a DIP subset with respect to comparable evaluations. More complete details are presented in [15].

**Figure 1:**
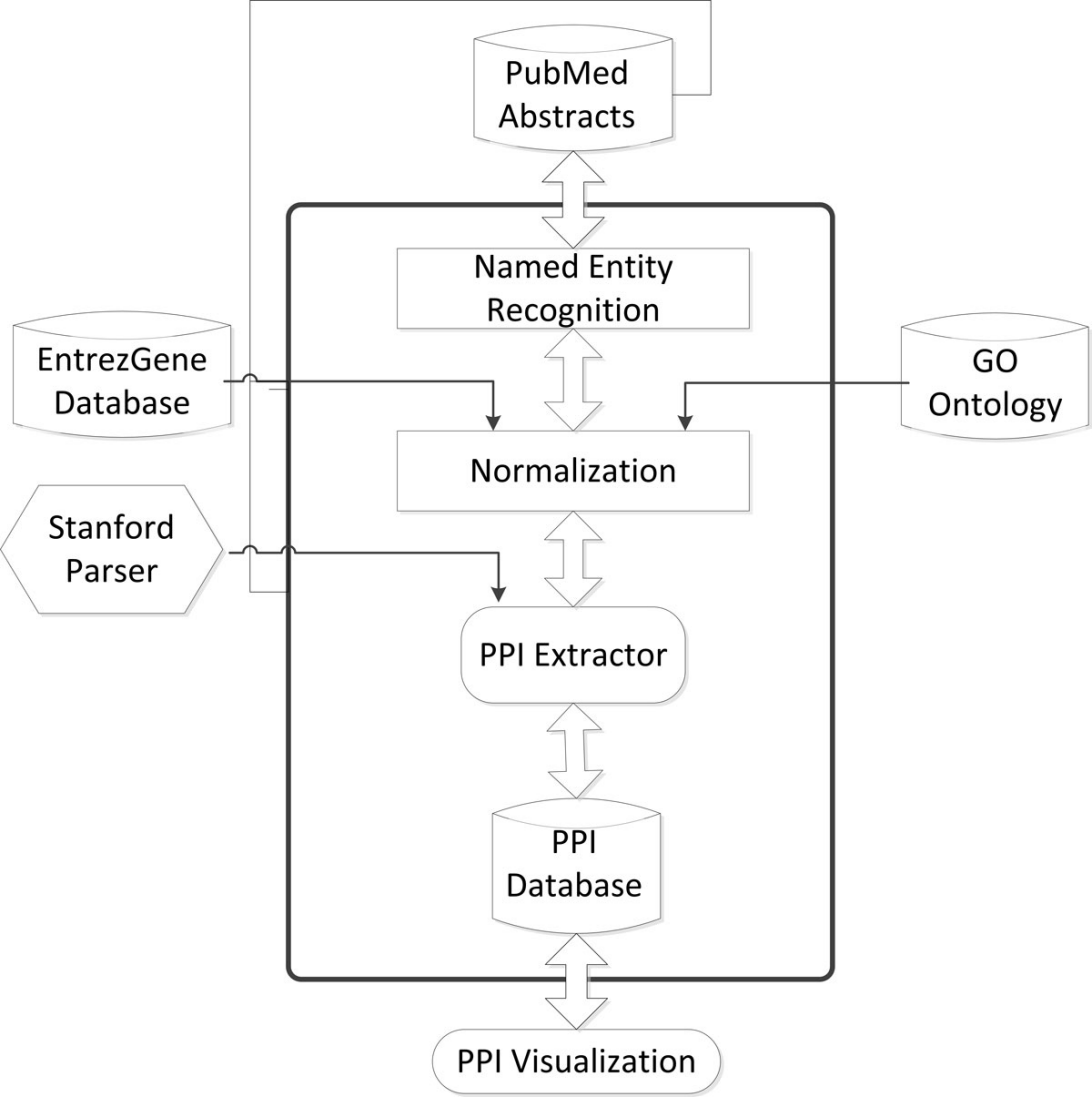
**The architecture of PPIExtractor**.

PPIExtractor contains four modules: (i) Named Entity Recognition (NER) module which aims to identify the protein names in the biomedical literature; (ii) Normalization module which determines the unique identifier of proteins identified in NER module; (iii) PPI extraction module which extracts the PPI information in the biomedical literature and (iv) PPI visualization module which displays the extracted PPI information in the form of a graph. Figure 1 shows the architecture of PPIExtractor.

The biomedical literature PPI data we used is 127,217 PubMed abstracts downloaded from PubMed website (http://www.ncbi.nlm.nih.gov/pubmed) with the query string "((Saccharomyces cerevisiae) OR yeast) AND protein". 126,165 protein interactions were extracted from these abstracts by the PPIExtractor system.

Most of the protein names in the PPI databases are systematic names for nuclear-encoded ORFs begin with the letter 'Y' (for 'Yeast') while those in PubMed abstracts are not. Therefore, we built a yeast protein alias name list with about 6,000 entries from the UniProt website (http://www.uniprot.org/uniprot/?query=yeast&sort=score). The list is used to convert the protein names in PubMed abstracts to systematic names for nuclear-encoded ORFs. In our method, a PPI can be added into a PPI dataset only if the two proteins in the PPI already exist in the PPI dataset.

### Yeast PPI datasets

As in [6], five different yeast PPI datasets in our experiments were used to verify the effectiveness of our method, including three high-throughput experimental datasets (Gavin, Krogan-core and Krogan-extended), a computationally derived network that integrates the results of these studies (Collins), and a compendium of all known yeast protein-protein interactions (BioGRID). The Gavin data set was obtained by considering all PPIs with a socio-affinity index larger than five, proposed by the original authors. The Krogan data set was used in two variants: the core data set and the extended data set. The core data set contained only highly reliable interactions, whose probability > 0.273. The extended data set contained more interactions with less reliability, whose probability > 0.101. The Collins data set was retained the top 9,074 interactions according to their purification enrichment score, as suggested in the original paper. The BioGRID data set was downloaded from version 3.1.77 and contained all physical interactions that involve yeast proteins only. The details of the interaction datasets are shown in Table 1. Self-interactions and isolated proteins were filtered from all the datasets.

**Table 1:** Properties of the five yeast PPI datasets used in the experiments

Datasets	Number of proteins	Number of interactions
Collins	1622	9074
Gavin	1855	7669
Krogan-core	2708	7123
Krogan-extended	3672	14317
BioGRID	5640	59748

### Integration of the extracted PPIs into the PPI datasets

**Figure 2:**
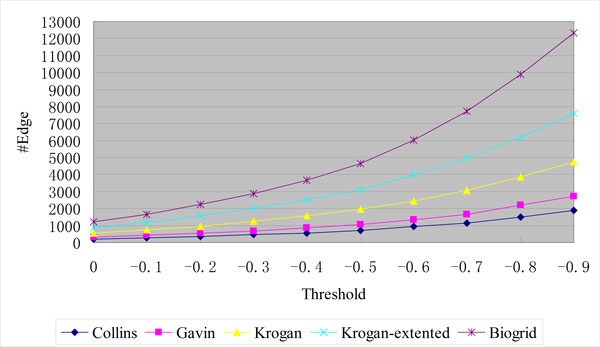
**The amounts of the PPIs added into the original PPI datasets**.

Each extracted PPI is assigned a weight by PPIExtractor which represent the reliability of the PPI. In our method, a certain amount of PPIs with the weights higher than a threshold can be integrated into the PPI datasets. Since BioGRID is an unweighted dataset, the weights of these PPIs are discarded. For the weighted datasets, Gavin, Krogan-core and Krogan-extended and Collins, the weights of these PPIs are adjusted proportionately to the ones in the PPI datasets which are usually calculated using complicated machine learning approaches that operate on the original noisy experimental datasets to reflect the reliability of the PPI [6]. In addition, we integrate a PPI with the weight equal to or higher than a threshold into the PPI dataset only if both two proteins in the PPI already exist in the PPI dataset. As shown in Figure 2, since the BioGRID dataset has the most proteins (5,460), the most PPIs are integrated into it: with the threshold -0.6, 6,025 PPIs are integrated into it. The amounts of the PPIs added into the PPI datasets with different thresholds are shown in Table 2.

**Table 2:** The amounts of the PPIs added into the original PPI datasets with different thresholds

Threshold	Collins	Gavin	Krogan- core	Krogan- extended	BioGRID
0	201	318	547	881	1210
-0.1	278	427	742	1192	1665
-0.2	354	551	964	1560	2232
-0.3	454	684	1245	1994	2865
-0.4	569	849	1560	2515	3654
-0.5	725	1046	1952	3128	4651
-0.6	926	1324	2457	3962	6025
-0.7	1149	1672	3071	4957	7715
-0.8	1505	2190	3871	6189	9894
-0.9	1892	2725	4714	7597	12320

### Protein complex detection methods

In our experiments, a state-of-the-art complex detection method, ClusterONE [6], was used to evaluate our method's effectiveness on PPI datasets for protein complex detection. The ClusterONE is a method for detecting potentially overlapping protein complexes from protein interaction network. The algorithm uses a greedy growth process to find groups in a protein interaction network. The main algorithm consists of three steps: first, it grows groups with high cohesiveness from selected seed proteins. Second, it merges highly overlapping pairs of locally optimal cohesive groups. Last, the complex candidates that contain less than three proteins or whose densities are below a given threshold are discarded. Experimental results show that ClusterONE outperforms the other approaches both on weighted and unweighted PPI networks, matching more complexes with a higher accuracy and providing a better one-to-one mapping with reference complexes in almost all the data sets.

In addition, we also evaluated the effectiveness of our method on three other complex detection algorithms proposed in recent years, i.e. CMC, COACH and RRW. CMC is a clique based method that uses a protein-protein interaction iteration method to update the network [2]. COACH is based on the core-attachment architecture developed by Gavin et al.[7], and selects some subgraph as the core structure first, and then adds the attachment to the core to construct a complex. The RRW algorithm derives complexes from results of repeated restarted random walks on the graph of protein-protein interactions [5]. For each algorithm, its parameters are set as those described in [6] which have been optimized to yield the best possible results as measured by the maximum matching ratio on the gold standards.

## Results and discussion

### Gold standard protein complexes

Like [6], the MIPS catalog of protein complexes [19] (18 May 2006) and the Gene Ontology (GO)-based protein complex annotations from SGD [20] (11 Aug 2010) were used as our gold standards. To avoid selection bias, all MIPS categories containing at least three and at most 100 proteins as protein complexes are considered. MIPS category 550 and all its descendants, as these categories correspond to unconfirmed protein complexes that were predicted by computational methods.

For SGD, GO annotations are maintained [21] for all yeast proteins. The complexes were derived from proteins annotated by descendant terms of the Gene Ontology term 'protein complex' (GO:0043234). Annotations with modifiers such as 'NOT' or 'colocalizes_with' and annotations supported by 'IEA' evidence code only were ignored. The details of the gold standard protein complex datasets are shown in Table 3.

**Table 3:** Details of the gold standard protein complex datasets

	MIPS	SGD
Number of complexes	203	323
The max size of complexes	95	55
The min size of complexes	3	1
The average size of complexes	12.5	5.4

### Evaluation metrics

Like [6], we used three independent quality measures to assess the similarity between a set of predicted complexes and a set of reference complexes. The first measure is the fraction of pairs between predicted and reference complexes with an overlap scoreω larger than 0.25. The overlap score between two protein sets A and B is defined as follows:

(1) $$NA\left(A,B\right)=\frac{|VA\cap VB{|}^{2}}{\left|VA|\times |VB\right|}$$

The threshold of 0.25 is chosen because it represents the case when the intersection is at least half of the complex size if the two complexes being compared are equally large.

The second measure we used is the geometric accuracy as introduced by Broh´ee and van Helden [22], which is the geometric mean of two other measures, namely the clustering-wise sensitivity (*Sn*) and the clustering-wise positive predictive value (*PPV*). Let n be the number of the benchmark complexes and m be the number of the predicted complexes. Construct a confusion matrix *T*, and let $T_{ij}$ denote the number of proteins that are found both in reference complex *i *and predicted complex *j. Sn *and *PPV *are defined as follows:

(2) $$Sn=\frac{\sum_{i=1}^{n}\underset{j}{\text{max}}\left\{T_{ij}\right\}}{\sum_{i=1}^{n}N_{i}}$$

(3) $$PPV=\frac{\sum_{j=1}^{m}\underset{i}{\text{max}}\left\{T_{ij}\right\}}{\sum_{i=1}^{m}T_{.j}}$$

Here, we define Ni is the number of proteins in the benchmark complex *i*, then $T_{.j}$is defined as:

(4) $$T_{.j}=\sum_{i=1}^{n}T_{ij}$$

Generally, a high *Sn *value indicates that the prediction has a good coverage of the proteins in the true complexes, whereas a high *PPV *value indicates that the predicted complexes are likely to be true complexes. So it is necessary to balance the two measures by introducing the geometric accuracy (*Acc*), which is simply the geometric mean of the clustering-wise sensitivity and the positive predictive value:

(5) $$Acc=\sqrt{Sn\times PPV}$$

The third measure we used is the maximum matching ratio (MMR) which was introduced in [6]. This measure is based on a maximal one-to-one mapping between predicted and standard complex. Let *R *as the standard complex, and *P *as the predicted complex. An edge connects a standard complex and a predicted complex if their neighborhood affinity score is larger than zero. Given *n *standard complexes and *m *predicted complexes, let *j *be the member of the predicted complexes, *MMR *then defined as follows:

(6) $$MMR=\frac{\sum_{i=1}^{n}\underset{j}{\text{max}}NA\left(i,j\right)}{n}$$

The geometric accuracy measure explicitly penalizes predicted complexes that do not match any of the reference complexes. However, gold standard sets of protein complexes are often incomplete [23]. As a consequence, predicted complexes not matching any known reference complexes may still exhibit high functional similarity or be highly co-localized, and therefore they could still be prospective candidates for further in-depth analysis. In other words, a predicted complex that does not match a reference complex is not necessarily an undesired result, and optimizing for the geometric accuracy measure might prevent us from detecting novel complexes from a PPI dataset. The maximum matching ratio sidesteps this problem by dividing the total weight of the maximum matching with the number of reference complexes. Therefore, in the performance comparison, the MMR is used as the main metric, and the Acc is only used as an auxiliary one.

### The performances of ClusterONE on PPI datasets

**Figure 3:**
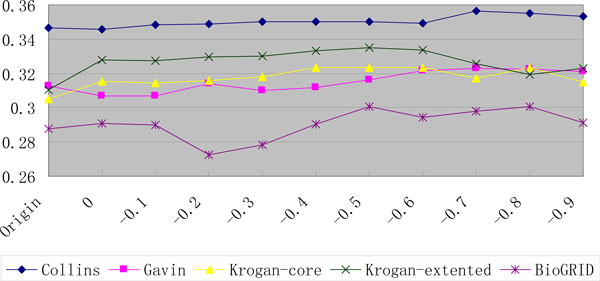
**The MMR performances of ClusterONE on PPI datasets using the MIPS gold standard**.

First, we tested ClusterONE on the Collins, Gavin, Krogan-core, Krogan-extended and BioGRID dataset. Tables 4, 5 and 6 contain the results of Accuracy, MMR and fraction of matched complexes when the MIPS dataset was used as the gold standard, respectively. Figure 3 depicts the MMR performances of ClusterONE on PPI datasets using the MIPS gold standard, which show that, in most cases, better performance of ClusterONE can be achieved when the PPIs extracted from literature are added into the original PPI datasets. When the PPIs with weights larger than or equal to threshold -0.6 are added, ClusterONE achieves the highest average MMR improvement on all five PPI datasets: the average improvements of 2.938 and 3.976 percentage units in Accuracy and MMR over that on the original datasets are achieved on the new datasets. With the lower thresholds (-0.7 to -0.9), the MMR performance begin to decline. The reason is that the lower threshold means more less reliable PPIs are introduced, which will deteriorate the performance of complex detection algorithms.

**Table 4:** The Accuracy performances of ClusterONE on PPI datasets using the MIPS gold standard

Threshold	Collins	Gavin	Krogan-core	Krogan-extended	BioGRID	Avg.Δ
Origin	0.4141	0.3727	0.3588	0.3661	0.4286	
0	0.4166	0.3738	0.3685	0.3807	0.4317	
-0.1	0.4168	0.3738	0.3705	0.3799	0.4301	
-0.2	0.4186	0.3812	0.3717	0.3818	0.4258	
-0.3	0.4168	0.3803	0.3732	0.3827	0.4302	
-0.4	0.419	0.3778	0.3754	0.383	0.4319	
-0.5	0.4209	0.3759	0.376	0.3822	0.4386	
**-0.6**	**0.4188**	**0.3813**	**0.374**	**0.3813**	**0.4409**	
-0.7	0.4222	0.3821	0.3746	0.3839	0.4415	
-0.8	0.4205	0.3847	0.3781	0.3818	0.4468	
-0.9	0.4193	0.3814	0.3779	0.3868	0.4393	
Δ(**-0.6**)	**1.13%**	**2.3%**	**4.24%**	**4.15%**	**2.87%**	**2.938%**

Δ(-0.6) denotes the MMR improvement with the threshold -0.6 over that on the original datasets. Avg.Δ denotes the average MMR improvement over that on the original datasets.

**Table 5:** The MMR performances of ClusterONE on PPI datasets using the MIPS gold standard

Threshold	Collins	Gavin	Krogan-core	Krogan-extended	BioGRID	Avg.Δ
Origin	0.3465	0.3125	0.3049	0.3103	0.2876	
0	0.3456	0.3069	0.3154	0.3277	0.2907	
-0.1	0.3482	0.3069	0.3143	0.3275	0.2901	
-0.2	0.3488	0.3142	0.316	0.3298	0.2726	
-0.3	0.3504	0.3102	0.3181	0.3302	0.2781	
-0.4	0.3502	0.3118	0.3232	0.3334	0.2902	
-0.5	0.3504	0.3163	0.3234	0.3348	0.3007	
**-0.6**	**0.3495**	**0.3216**	**0.3236**	**0.3338**	**0.2945**	
-0.7	0.3564	0.323	0.3172	0.3258	0.2978	
-0.8	0.3549	0.3226	0.3236	0.3195	0.3005	
-0.9	0.3534	0.3213	0.3149	0.3231	0.2913	
Δ(**-0.6**)	**0.87%**	**2.91%**	**6.13%**	**7.57%**	**2.40%**	**3.976%**

**Table 6:** The fraction of matched complexes with a given overlap score threshold (ω ≥ 0.25) of ClusterONE on PPI datasets using the MIPS gold standard

Threshold	Collins		Gavin		Krogan-core		Krogan-extended		BioGRID	
	
	#cluster	#matched	#cluster	#matched	#cluster	#matched	#cluster	#matched	#cluster	#matched
Origin	195	78	194	68	522	77	531	94	472	87
0	210	75	226	68	520	88	622	115	487	81
-0.1	208	77	226	68	522	88	619	117	488	81
-0.2	206	76	226	71	516	87	615	107	503	75
-0.3	214	78	229	72	504	80	610	110	509	75
-0.4	212	77	233	73	503	87	612	101	523	86
-0.5	211	74	241	72	499	87	613	103	554	87
**-0.6**	**206**	**78**	**242**	**74**	**493**	**87**	**599**	**103**	**577**	**87**
-0.7	209	81	253	77	484	88	602	102	601	89
-0.8	206	81	245	78	471	90	617	99	640	90
-0.9	206	79	242	80	470	88	608	98	679	90

**Figure 4:**
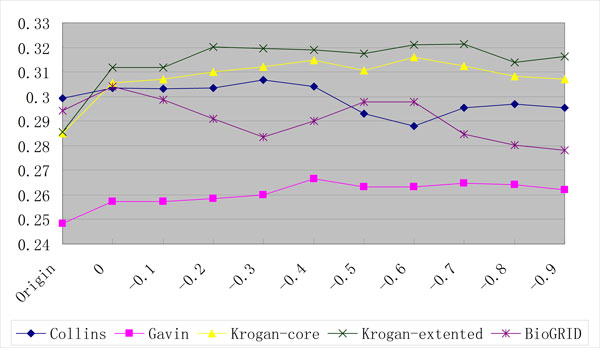
**The MMR performances of ClusterONE on PPI datasets using the SGD gold standard**.

The similar results were obtained when the SGD dataset was used as the gold standard as shown in Figure 4 and Tables 7, 8 and 9. Compared with the original datasets, the average improvements of 2.356 and 5.416 percentage units in Accuracy and MMR are achieved on the new networks with the threshold -0.6.

**Table 7:** The Accuracy performances of ClusterONE on PPI datasets using the SGD gold standard

Threshold	Collins	Gavin	Krogan-core	Krogan-extended	BioGRID	Avg. Δ
Origin	0.5505	0.5127	0.5501	0.554	0.6020	
0	0.5512	0.5116	0.5686	0.5732	0.6171	
-0.1	0.5457	0.5116	0.5704	0.5694	0.6197	
-0.2	0.5501	0.5162	0.5686	0.5724	0.6144	
-0.3	0.5518	0.5211	0.5692	0.5715	0.6101	
-0.4	0.5520	0.5191	0.573	0.5709	0.6077	
-0.5	0.5487	0.5183	0.5681	0.5682	0.6073	
**-0.6**	**0.5501**	**0.5261**	**0.5669**	**0.5712**	**0.6202**	
-0.7	0.556	0.526	0.5716	0.5746	0.6126	
-0.8	0.5579	0.5253	0.568	0.5701	0.6104	
-0.9	0.5621	0.5244	0.568	0.5725	0.6097	
Δ(**-0.6**)	**0**	**2.61%**	**3.05%**	**3.10%**	**3.02%**	**2.356%**

**Table 8:** The MMR performances of ClusterONE on PPI datasets using the SGD gold standard

Threshold	Collins	Gavin	Krogan-core	Krogan-extended	BioGRID	Avg.Δ
Origin	0.2994	0.2483	0.2849	0.2856	0.2942	
0	0.3035	0.2574	0.3057	0.3117	0.3040	
-0.1	0.3033	0.2574	0.3072	0.3118	0.2987	
-0.2	0.3034	0.2584	0.3099	0.3202	0.2911	
-0.3	0.3068	0.26	0.312	0.3195	0.2836	
-0.4	0.3042	0.2665	0.3147	0.3189	0.2900	
-0.5	0.2931	0.2632	0.3107	0.3176	0.2977	
**-0.6**	**0.2879**	**0.2633**	**0.3159**	**0.3211**	**0.2977**	
-0.7	0.2953	0.2648	0.3123	0.3214	0.2847	
-0.8	0.297	0.264	0.3082	0.3139	0.2802	
-0.9	0.2955	0.2622	0.3071	0.3162	0.2782	
Δ(**-0.6**)	**-3.84%**	**6.4%**	**10.9%**	**12.43%**	**1.19%**	**5.416%**

**Table 9:** The fraction of matched complexes with a given overlap score threshold (ω ≥ 0.25) of ClusterONE on PPI datasets using the SGD gold standard

Threshold	Collins		Gavin		Krogan-core		Krogan-extended		BioGRID	
	
	#cluster	#matched	#cluster	#matched	#cluster	#matched	#cluster	#matched	#cluster	#matched
Origin	195	105	194	99	522	168	531	175	472	157
0	210	112	226	107	520	187	622	202	487	156
-0.1	208	113	226	107	522	189	619	204	488	154
-0.2	206	111	226	107	516	187	615	195	503	148
-0.3	214	112	229	110	504	184	610	200	509	140
-0.4	212	112	233	114	503	185	612	192	523	145
-0.5	211	110	241	112	499	184	613	194	554	147
**-0.6**	**206**	**110**	**242**	**110**	**493**	**183**	**599**	**193**	**577**	**153**
-0.7	209	114	253	114	484	182	602	198	601	143
-0.8	206	112	245	114	471	175	617	184	640	141
-0.9	206	111	242	117	470	175	608	194	679	142

### The performances of other algorithms on PPI datasets

The performances of three other complex detection algorithms proposed since 2009 (i.e. COACH, CMC and RRW) on these yeast PPI datasets are shown in Tables 10 and 11. Like ClusterONE, these algorithms achieve the best performance with the threshold -0.6 on these yeast PPI datasets except on BioGRID: in term of MMR, COACH, CMC and RRW achieve 12.51, 19.85 and 4.2 percentage unit average improvements over those on the original datasets using the MIPS gold standard, respectively, while the average improvements are 12.41, 15.59 and 5.85 percentage units using the SGD gold standard, respectively.

**Table 10:** The performances of various protein complex detection algorithms on PPI datasets using the MIPS gold standard

		Collins	Gavin	Krogan-core	Krogan-extended	BioGRID	Avg.Δ
C O A C H	Accuracy	0.4384	0.3706	0.3164	0.3168	0.4120	
	Accuracy(-0.6)	0.4325	0.3783	0.3391	0.3386	0.4164	
	Δ(-0.6)	**-1.34%**	**2.08%**	**7.17%**	**6.88%**	**1.07%**	**3.17%**
	Accuracy(0)					0.4196	
	Δ(0)					**1.84%**	
	MMR	0.3390	0.3164	0.2630	0.2719	0.3221	
	MMR(-0.6)	0.3671	0.3368	0.3202	0.3372	0.3287	
	Δ(-0.6)	**8.29%**	**6.45%**	**21.75%**	**24.02%**	**2.05%**	**12.51%**
	MMR(0)					0.3296	
	Δ(0)					**2.33%**	
C M C	Accuracy	0.382	0.3329	0.2945	0.2956	0.3000	
	Accuracy(-0.6)	0.3922	0.3371	0.3209	0.317	0.3056	
	Δ(-0.6)	**2.67%**	**1.26%**	**8.96%**	**7.24%**	**1.87%**	**4.4%**
	Accuracy(0)					0.3085	
	Δ(0)					**2.83%**	
	MMR	0.2593	0.2859	0.1821	0.2039	0.0680	
	MMR(-0.6)	0.2935	0.3118	0.2527	0.2752	0.0702	
	Δ(-0.6)	**13.19%**	**9.06%**	**38.77%**	**34.97%**	**3.24%**	**19.85%**
	MMR(0)					0.0719	
	Δ(0)					**5.74%**	
R R W	Accuracy	0.3382	0.3339	0.2886	0.2975	0.3409	
	Accuracy(-0.6)	0.3433	0.3387	0.3095	0.3139	0.3361	
	Δ(-0.6)	**1.51%**	**1.44%**	**7.24%**	**5.51%**	**-1.41%**	**2.86%**
	Accuracy(0)					0.3447	
	Δ(0)					**1.11%**	
	MMR	0.3148	0.2959	0.2479	0.2549	0.2759	
	MMR(-0.6)	0.3198	0.3058	0.2854	0.2787	0.2527	
	Δ(-0.6)	**1.59%**	**3.35%**	**15.13%**	**9.34%**	**-8.41%**	**4.2%**
	MMR(0)					0.2794	
	Δ(0)					**1.27%**	

MMR(-0.6) denotes the MMR value when with the threshold -0.6; Δ(-0.6) denotes the MMR improvement when with the threshold -0.6 over that on the original datasets. MMR(0) denotes the MMR value when with the threshold 0; Δ(0) denotes the MMR improvement when with the threshold 0 over that on the original datasets.

**Table 11:** The performances of various protein complex detection algorithms on PPI datasets using the SGD gold standard

		Collins	Gavin	Krogan-core	Krogan-extended	BioGRID	Avg.Δ
C O A C H	Accuracy	0.4930	0.5042	0.4515	0.4519	0.4750	
	Accuracy(-0.6)	0.4937	0.5085	0.4804	0.4852	0.4766	
	Δ(-0.6)	**0.14%**	**0.85%**	**6.40%**	**7.37%**	**0.34%**	**3.02%**
	Accuracy(0)					0.4779	
	Δ(0)					**0.61%**	
	MMR	0.2747	0.2614	0.2466	0.2538	0.3000	
	MMR(-0.6)	0.3056	0.2804	0.3000	0.3076	0.3020	
	Δ(-0.6)	**11.25%**	**7.27%**	**21.65%**	**21.20%**	**0.67%**	**12.41%**
	MMR(0)					0.2993	
	Δ(0)					**-0.23%**	
C M C	Accuracy	0.4635	0.4518	0.4179	0.4393	0.3203	
	Accuracy(-0.6)	0.4819	0.4587	0.4579	0.4746	0.3159	
	Δ(-0.6)	**3.97%**	**1.53%**	**9.57%**	**8.04%**	**-1.37%**	**4.35%**
	Accuracy(0)					0.3283	
	Δ(0)					**2.50%**	
	MMR	0.2006	0.2378	0.1552	0.1724	0.0583	
	MMR(-0.6)	0.2273	0.2545	0.2135	0.2262	0.0518	
	Δ(-0.6)	**13.31%**	**7.02%**	**37.56%**	**31.21%**	**-11.15%**	**15.59%**
	MMR(0)					0.0629	
	Δ(0)					**7.89%**	
R R W	Accuracy	0.5022	0.498	0.4676	0.469	0.5188	
	Accuracy(-0.6)	0.5098	0.5009	0.4855	0.4893	0.5062	
	Δ(-0.6)	**1.51%**	**0.58%**	**3.83%**	**4.33%**	**-2.43%**	**1.56%**
	Accuracy(0)					0.528	
	Δ(0)					**1.77%**	
	MMR	0.2763	0.2636	0.2414	0.2476	0.2681	
	MMR(-0.6)	0.2901	0.2698	0.2771	0.2773	0.2550	
	Δ(-0.6)	**4.99%**	**2.35%**	**14.79%**	**12.00%**	**-4.89%**	**5.85%**
	MMR(0)					0.2822	
	Δ(0)					**5.26%**	

On the BioGRID dataset, the performances of these algorithms decrease with the threshold -0.6: in term of MMR, there is an 8.41 percentage unit decrease in the performance of the RRW algorithm using the MIPS gold standard while there are 11.15 and 4.89 percentage unit decreases in the performance of the CMC and RRW algorithms using the SGD gold standard, respectively. Through the analysis of the results, we found that these algorithms obtain more clusters on BioGRID with the threshold -0.6 than on the original BioGRID. However, many of them are not matched one, i.e. they can not match with any complex in the gold standards, which deteriorates the performances of the complex detection algorithms.

**Figure 5:**
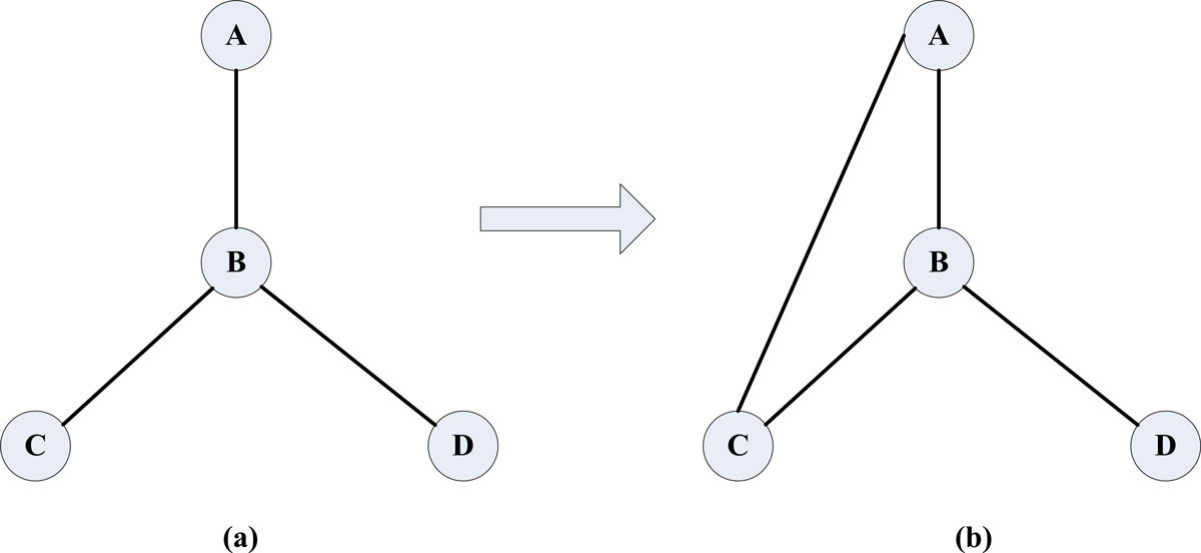
**An example of a candidate complex**. (a) before the PPI integration and (b) after the PPI integration.

The reason behind it is that, in our method, a PPI with the weight equal to or higher than a threshold is integrated into the PPI dataset only if both two proteins in the PPI already exist in the PPI dataset. Since the BioGRID dataset includes the most proteins (5,460), the most PPIs are integrated into it as shown in Figure 2: with the threshold -0.6, 6,025 PPIs are integrated into it while the numbers are 926, 1,324, 2,457 and 3,962 for Collins, Gavin, Krogan-core, Krogan-extended, respectively. In fact, according to [6], the BioGRID network is structurally very different from the other four datasets, and particularly it shows an unexpectedly high fraction of star-like structures. If many candidate complexes with star-like structures are predicted, the effectiveness of the complex detection algorithms may be hampered. The reason is that these complexes usually have low density values (where the density of a complex with n proteins is defined as the total weight of its internal edges, divided by n * (n − 1)/2 and, in the unweighted BioGRID dataset, the total weight of the complex is the number of its internal edges; an example is shown in Figure 5a) and a considerable number of real complexes form a clique in the interaction graph and have high density values though there are many other topological structures that may represent a complex on a PPI graph [24]. For example, the experimental results in [6] show that the performance of various protein complex detection algorithms on BioGRID is the worst among all PPI databases. In these cases the authors of [6] recommended that use higher value for the density threshold in order to discard trivial clusters. Given an unweighted network, ClusterONE automatically tests the value of the transitivity and sets the density threshold to either 0.5 or 0.6 (for the BioGRID dataset it uses 0.6).

On a dataset like BioGRID, many candidate complexes with star-like structures and low density values should have been discarded based on the density threshold by complex detection algorithms. However, when the PPI data from literature are integrated, many such candidate complexes will be retained since the density values of these complexes are increased with the inclusion of new PPI data. As shown in the example of Figure 5, a candidate complex with star-like structure (Figure 5a) will be discarded since its density is 0.5 while the density threshold. However, when the edge between protein A and C is added (Figure 5b), the complex's density increases to 0.67 and it will be retained by ClusterONE (the density threshold 0.6).

**Figure 6:**
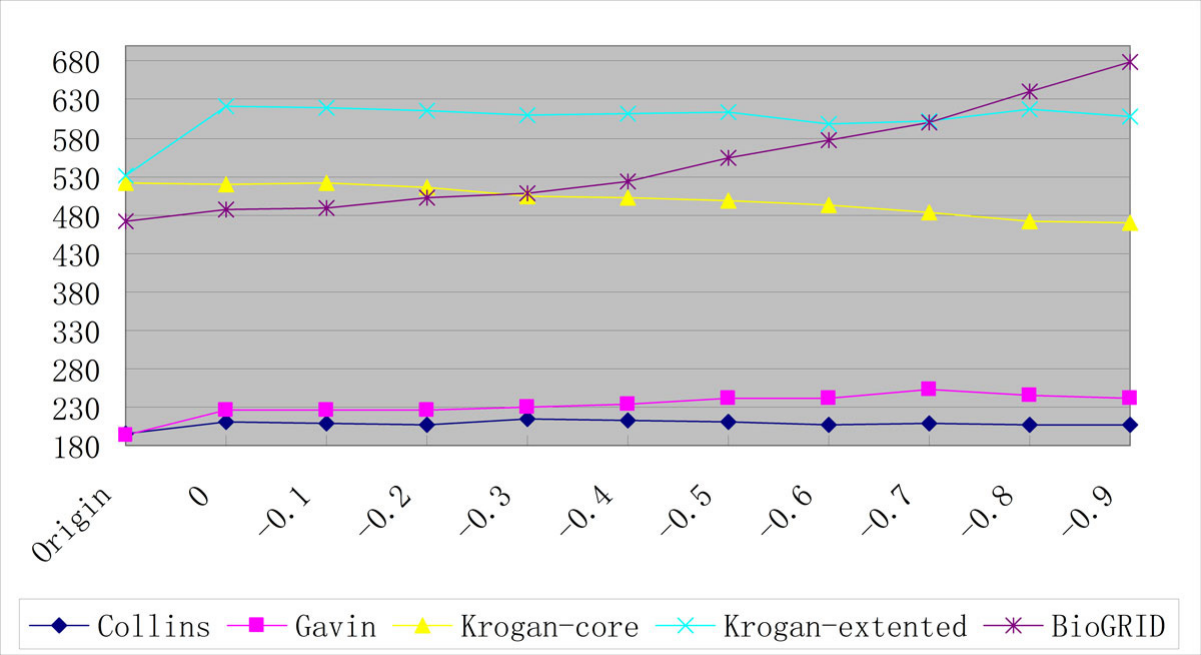
**The numbers of the complexes detected by ClusterONE on PPI datasets with different thresholds**.

**Figure 7:**
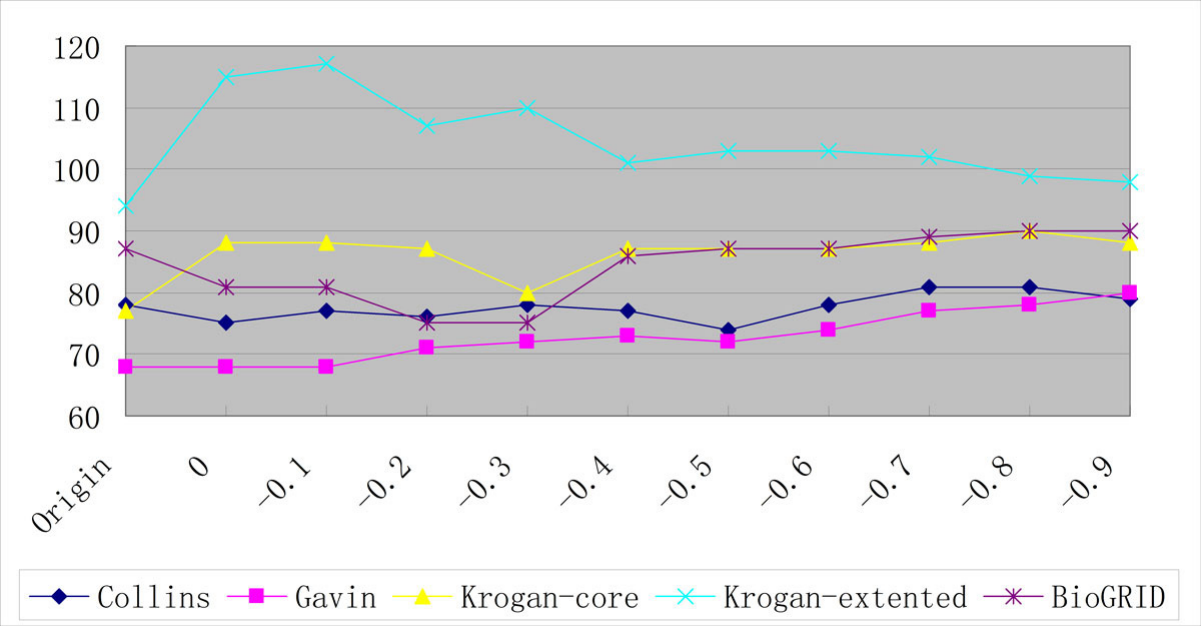
**The numbers of the matched complexes detected by ClusterONE on PPI datasets with different thresholds using the MIPS gold standard**.

**Figure 8:**
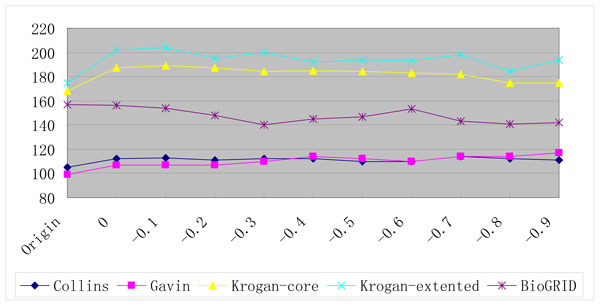
**The numbers of the matched complexes detected by ClusterONE on PPI datasets with different thresholds using the SGD gold standard**.

This assumption can be supported by the following fact: with the threshold -0.6, a total of 6,025 PPIs are integrated into the BioGRID dataset and a total of 105 detected complexes by ClusterONE are increased (from 472 to 577). Since many of them can not match with any complex in the gold standards, the performance is deteriorated. As can be seen from Figures 6, 7 and 8, with the increase of the threshold, the number of the detected complexes detected by ClusterONE on BioGRID dataset keeps increasing while the number of the matched complexes remains almost the same and, in some cases, even decreases. while on another dataset with large size, Krogan extended, with the threshold -0.6, a total of 3,962 PPIs are integrated and only 68 detected complexes are increased (from 531 to 599). Even with the threshold -0.8, a total of 6,189 PPIs (the number is equivalent to the one on BioGRID with the threshold -0.6) are integrated and 86 detected complexes are increased (from 531 to 617). As can be seen from Figures 6, 7 and 8, when the PPI data with the threshold 0 are included, the numbers of the detected complexes and matched complexes by ClusterONE on Krogan extended dataset both increase. With the further increase of the threshold, like on BioGRID, the number of the matched complexes remains almost the same and, in some cases, even decreases. However, the number of the detected complexes also decreases while on BioGRID it keeps ever increasing, which especially deteriorates the performance of ClusterONE on BioGRID.

**Figure 9:**
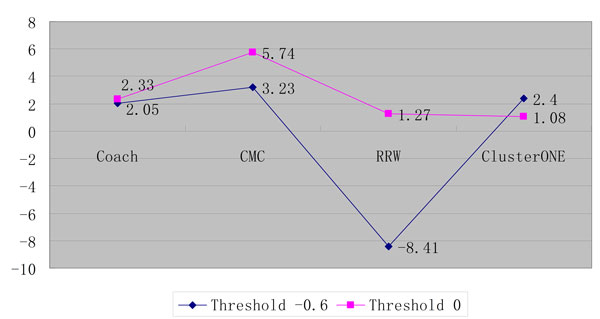
**The performance comparison of various protein complex detection algorithms on BioGRID between the threshold -0.6 and 0 using MIPS as gold standard**.

**Figure 10:**
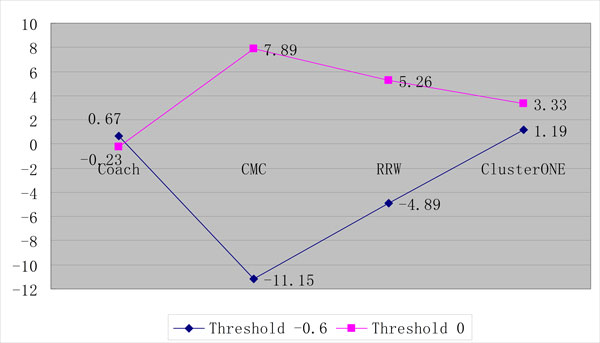
**The performance comparison of various protein complex detection algorithms on BioGRID between the threshold -0.6 and 0 using SGD as gold standard**.

On the other hand, we found if the threshold is set to 0 and less PPIs (1,210) are integrated into BioGRID, much better performance can be achieved using any gold standard (MIPS and SGD) as shown in Figures 9 and 10.

Therefore, with the databases with the low transitivity like BioGRID, the threshold should be set to higher to ensure less PPIs are integrated into the databases, and, in other cases, the threshold can be set to -0.6. In this way, the performances of protein complex detection algorithms can be improved through the integration of PPI datasets and the PPI data extracted from literature.

## Conclusions

Protein complexes are important for understanding principles of cellular organization and function. High-throughput experimental techniques have produced a large amount of protein interactions, making it possible to predict protein complexes from protein-protein interaction networks. On the other hand, the rapidly growing biomedical literature provides a significantly large, readily available and relatively accurate source of interaction data, which can be integrated into the protein network for better protein complex detection performance. In this paper, we present an approach of improving protein complex detection methods with integrated PPI data from biomedical literature. The approach applies PPIExtractor to extract PPI data from biomedical literature, which are then integrated into the protein network for protein complex detection. The experimental results of ClusterONE on five yeast PPI datasets show the effectiveness of our method: compared with the original networks, the average improvements of 3.976 and 5.416 percentage units in MMR are achieved on the new networks using the MIPS and SGD gold standards, respectively. In addition, our method also proves to be effective for three other algorithms proposed in recent years, CMC, COACH and RRW.

Through the analysis of the experimental results, we found the choice of the threshold usually can be set to -0.6. However, for the databases with the low transitivity like BioGRID, the threshold should be set to higher. In this way, the performances of the state-of-the-art protein complex detection algorithms can be improved through the integration of the existed PPI datasets and the PPI data extracted from literature.

A rapidly growing literature corpus ensures that PPI data is a readily-available resource for nearly every studied organism, particularly those with small protein interaction databases. PPI data provides a significantly large and readily available source of interaction data which, together with the guidelines and results reported here, will prove valuable especially for organisms in which protein-protein interaction data is sparse.

## Competing interests

The authors declare that they have no competing interests.

## Authors' contributions

ZHY conceived of the study, carried out its design and drafted the manuscript. FYY participated in the design of the study and performed the experiments. HFL and JW participated in its design and coordination, and helped to draft the manuscript. All authors read and approved the final manuscript.

## Declarations

Publication of this article was funded by the following grants: the Natural Science Foundation of China (grant no. 61070098, 61272373 and 61340020), Trans-Century Training Programme Foundation for the Talents by the Ministry of Education of China (grant no. NCET-13-0084) and the Fundamental Research Funds for the Central Universities (grant no. DUT13JB09 and DUT14YQ213).

This article has been published as part of *BMC Medical Genomics *Volume 7 Supplement 2, 2014: IEEE International Conference on Bioinformatics and Biomedicine (BIBM 2013): Bioinformatics in Medical Genomics. The full contents of the supplement are available online at http://www.biomedcentral.com/bmcmedgenomics/supplements/7/S2.
